# Durability of a New Thermal Aerogel-Based Rendering System under Distinct Accelerated Aging Conditions

**DOI:** 10.3390/ma14185413

**Published:** 2021-09-18

**Authors:** Joana Maia, Marco Pedroso, Nuno M. M. Ramos, Inês Flores-Colen, Pedro F. Pereira, Luís Silva

**Affiliations:** 1 Institute of R&D in Structures and Construction (CONSTRUCT), Laboratory of Building Physics (LFC), Faculty of Engineering (FEUP), University of Porto, 4200-465 Porto, Portugal; nmmr@fe.up.pt (N.M.M.R.); fpfp@fe.up.pt (P.F.P.); 2 CERIS, Civil Engineering Research and Innovation for Sustainability, Instituto Superior Técnico (IST), Universidade de Lisboa (UL), 1049-001 Lisboa, Portugal; marco.pedroso@tecnico.ulisboa.pt (M.P.); ines.flores.colen@tecnico.ulisboa.pt (I.F.-C.); 3 Saint-Gobain Weber, 3800-055 Aveiro, Portugal; luis.silva@saint-gobain.com

**Keywords:** aerogel, cement-based mortar, thermal render, durability, accelerated aging, experimental test

## Abstract

The widespread application of innovative thermal enhanced façade solutions requires an adequate durability evaluation. The present work intends to assess the durability of a new aerogel cement-based rendering system through the adaptation of different accelerated aging cycles, such as heating–freezing, freeze–thawing, and heat–cold. Several mechanical properties and also capillary and liquid water absorptions were tested for uncoated and coated specimens. A decrease in the mechanical strength, especially after freeze–thaw cycles, was observed. However, the water action promoted the late hydration of the cement paste contributing to the densification of the matrix and, consequently, the increase of the adhesive strength. Additionally, a decrease in the dynamic modulus of elasticity and an increase in the Poisson’s ratio were observed after aging, which indicates a higher capacity of the render to adapt to substrate movements, contributing to a reduction of cracking.

## 1. Introduction

To obtain more durable and, consequently, more sustainable buildings, the improvement of materials, with a particular emphasis on envelopes, takes a very important role by increasing their performance, enhancing the service life, and lowering the production and accumulation of waste [1]. The development of new construction materials, with emphasis on insulation, should be focused on the relation between thermal and mechanical properties of materials, as well as their composition [2]. Regarding the strict European criteria for the U-value (thermal transmittance) of building envelopes [3], several studies on the development of thermal enhanced building materials and components have been performed, with a special interest in thermal mortars [4,5,6,7]. Additionally, the incorporation of nanoparticles in mortars could improve their durability compared to traditional building materials [8]. In the specific case of aerogel-based renders, the incorporation of these nanomaterials allowed a reduction in building energy consumption [9,10,11] and wall U-value [12], since they present specific properties (low density and high porosity [13,14] due to its mesoporous structure) that leads to a very low thermal conductivity [15,16,17]. In addition, it is possible to include aerogel in different materials and components [18,19,20,21]. Although the wide use of aerogel in thermal insulation plasters [22,23], the possibility of external applications implies that a reliable study of their durability regarding exterior climatic agents is of great importance.

The hygrothermal and mechanical behavior and, consequently, the durability of mortars highly depend on their microstructure [24], such as the size, shape, and distribution of pores [25], which influences water permeability and absorption capacity [26,27]. These water transport mechanisms are responsible for how degradation agents enter the materials, which compromises the durability of materials with a high porosity since the permeability and water absorption tend to increase with the porosity [28,29]. The degradation of mortars are mainly caused by water, biological, mechanical, and human actions [30,31], which often occur combined [32,33]. The action of water and temperature is one of the most frequent combinations of degradation in rendering mortars. Cracking caused by the temperature gradient and, consequently, quicker water absorption is often observed in these materials [34].

To assess durability, the degradation mechanisms should be as close as possible to real climatic conditions, which is usually determined using accelerated aging tests.

Ihara et al. [35] assessed the durability of different sizes of silica aerogel granules for moisture and solar radiation action. Moisture aging comprised 300 cycles of temperature variations (35–65 °C) at 100% constant relative humidity. It was stated that aerogel should be protected from moisture action, since the thermal conductivity of the small and large granules increased by 8% and 11%, respectively. Solar radiation aging was carried out in two steps: first, 5 h of solar radiation (simulated through a metal halide global lamp) at 60 °C and 50% RH (relative humidity) and then an hour-long water (10 °C) spray. Visible changes after solar irradiation action were not found in the small aerogel granules that maintained a hydrophobic surface.

Berardi and Nosrati [36] tested different products containing aerogel (aerogel-enhanced plasters, blankets, fiberboards, and gypsum boards) using three accelerated aging procedures: elevated temperature (70 °C), freeze-thaw (daily cycle: 40 °C to −30 °C) and high moisture (45 °C and 70% relative humidity). It was highlighted that the thermal performance of the tested materials after aging was similar to that of the non-aged material, which indicates the capability of the aerogel-based materials to maintain good thermal behavior over time.

Pereira et al. [37] also characterized the physical and mechanical behavior of thermal mortars incorporating silica aerogel as an aggregate, exposed to elevated temperatures (20 °C to 250 °C). The observed worse performance after the exposure to elevated temperatures resulted from the incorporation of admixtures in the binder paste and not from the aerogel aggregate. In addition, the thermal properties were not substantially affected by the elevated temperatures; however, when exposed to temperatures above 100 °C, the mechanical behavior of the thermal mortars could be compromised due to internal damage and a consequent reduction in mechanical properties. However, durability assessments of this type of material are still scarce and without standard procedures. In addition, there is a gap in the evaluation of the system as a whole (aerogel-based render and protective coating).

This paper intends to discuss the long-term performance of aerogel cement-based rendering systems and contribute to the knowledge of the in-service performance of innovative solutions, complementing the hygrothermal performance analysis carried out by Maia et al. [38]. As such, the present work has the objective of assessing the durability of a new aerogel cement-based rendering system exposed to distinct environmental conditions, such as freeze–thaw, humidification, and heat–cold accelerated degradation mechanisms. Aging methods applicable to one-coat renders, multilayer systems, and thermal rendering systems will be adapted to assess the durability of the new aerogel cement-based rendering system, especially in terms of the hygrothermal and mechanical behavior. Since aerogel-based renders present a high open porosity and low mechanical strength [39], the study of the entire rendering system (thermal render and protective coating system) and the contribution of the protective layer is of great importance.

## 2. Materials and Methods

### 2.1. Experimental Methodology

**Figure 1:**
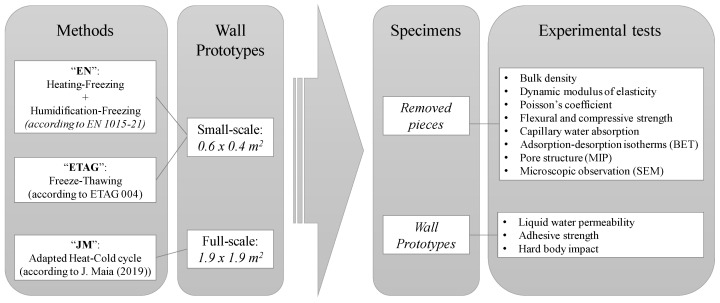
Experimental methodology.

The experimental methodology used in assessing the durability of a new aerogel cement-based rendering system was studied by Pedroso et al. [40] and is based on adapting existing methodologies. Figure 1 summarizes the experimental methodology, comprising the different specimens, accelerated aging methods, and corresponding durability tests.

**Figure 2:**
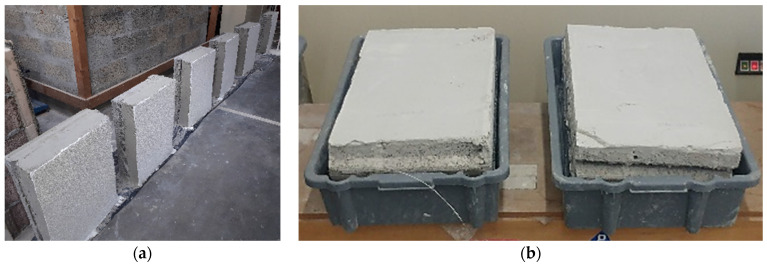
(**a**) Small-scale specimens (0.6 × 0.4 m^2^); (**b**) humidification/thawing procedure.

**Figure 3:**
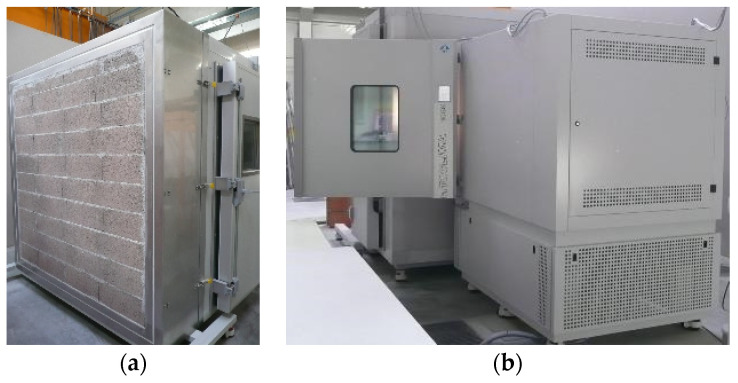
Climatic chamber Fitoclima 1000 EDTU: (**a**) portable rig, where the wall prototype (1.9 × 1.9 m^2^ full-scale) was built, coupled to the climatic chamber; (**b**) back view.

The accelerated aging procedures presented in EN(European Norm) 1015-21 (EN method) and ETAG (European Technical Approval Guideline) 004 (ETAG method) were applied to small-scale wall prototypes (0.6 × 0.4 m^2^), as shown in Figure 2a. The heat–cold cycle (JM (Joana Maia) method) developed by Maia et al. [41], specifically designed for thermal rendering systems, was applied to a full-scale wall prototype (1.9 × 1.9 m^2^/rig), as can be seen in Figure 3a. Except for the capillary water absorption test, which was performed in both removed pieces of aerogel cement-based render and rendering systems (render plus protective coating system), the remaining tests were performed only on the render. The wall prototypes consisted of the aerogel cement-based rendering system.

The EN aging method combines heating-freezing series—8 h of infrared radiation (60 ± 2 °C) and 15 h of freezing (−15 ± 1 °C)—with a humidification-freezing series—immersion for 8 h in water at room temperature and then 15 h of exposure to −15 ± 1 °C. Between the two series, the specimens were conditioned for 48 h at 20 ± 2 °C and 65 ± 5% relative humidity. The ETAG method was composed of 30 cycles of 8 h of humidification by immersing the surface of the specimen in water at 23 ± 2 °C and then 16 h of freezing at −20 °C. An infra-red (IR) lamp device (Solamagic, D-07937 Zeulenroda-Triebes, Duitsland, Germany) (2000 W) was used for the heating period and a deep-freeze cabinet (SMEG EL5 = SCO, 42016 Guastalla (RE), Italy) was used for freezing. Concerning the humidification/thawing procedure, a waterproof box was used to keep the rendered surface below a minimum 15 mm water depth (see Figure 2b).

The JM aging cycle consisted of a heat-cold cycle—−5 °C for 11 h and then 65 °C for 7 h. The temperature increased linearly and decreased between the two stages, which resulted in a complete daily cycle (24 h), and was repeated 30 times. This aging cycle was implemented in the wall/rig (see Figure 3a) which was coupled to a climatic chamber, Fitoclima 1000 EDTU (Aralab, 2635-047 Lisboa, Portugal) (see Figure 3b).

After the implementation of the accelerated aging methods, the specimens were conditioned to ambient conditions in the laboratory (about 20 ± 3 °C and 55 ± 5% relative humidity) for 30 days. All accelerated aging methods were carried out in the Laboratory of Building Physics (LFC) in the Faculty of Engineering of the University of Porto (FEUP).

### 2.2. Materials

#### 2.2.1. Mortar and Protective Coating System

In this research, the durability of the new aerogel cement-based rendering system [40,42] was tested under different accelerated aging cycles. This mortar presents a mixture of mineral binders, rheological agents, resins, and hydrophobic agents, among others, in its composition. The lightweight aggregate with thermal insulation properties is a commercially available hybrid supercritical silica aerogel in the form of granules [43], representing approximately 70% of the total volume of the mortar, which was incorporated. Twenty percent (m/m) corresponded to mineral binders, ~37% (m/m) or 70% (of the total volume) to silica aerogel hydrophobic granules, with the remaining proportion related to other components (e.g., lightweight filler) but without sand. This formulation was based on the work developed by Pedroso et al. [40]. The render was mechanically projected onto a wall built using lightweight concrete blocks.

The components of the protective coating system are commercially available and were composed of a basecoat [44], in which was embedded a fiberglass mesh and a finishing coating [45]. The total protective coating system, previously studied by Pedroso et al. [46], presented a thickness of ~5 mm, where the basecoat with the fiberglass was ~3 mm thick and the coating system was ~2 mm in thickness. This coating system was intended, not only to improve the mechanical resistance of the complete multi-layered solution but, at the same time, to significantly reduce the capillary water absorption of the render [46].

#### 2.2.2. Collection and Preparation of the Specimens

**Figure 4:**
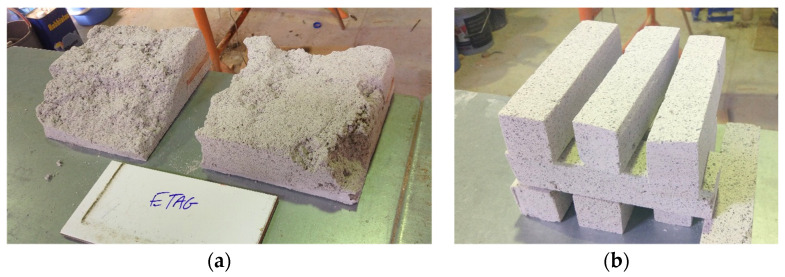
(**a**) Original pieces of the mortar with the protective coating on their surface after wall removal; (**b**) prismatic specimens after cutting from original pieces.

To obtain the several specimens required for the laboratory tests, the following steps were performed. Due to the characteristics of these rendering systems, it was possible to remove, through sawing, grinding, and cutting, large specimens, even when they were applied to wall surfaces. Therefore, several pieces of the mortar itself and mortar with the protective coating system were removed from the prototypes after the accelerated aging procedures (Figure 4a). Due to the intrusive process of the aging tests, no specimens were collected before this. After the very delicate processes of cutting and grinding, to avoid damaging the materials, different specimens were prepared for further testing. As an example, normalized prisms (160 × 40 × 40 mm^3^) are presented in Figure 4b.

For each accelerated aging method, several specimens were obtained. The normalized prismatic specimens allowed to characterize the bulk density, dynamic modulus of elasticity, Poisson’s coefficient, and flexural and compressive strengths. For the adhesive strength, cubic specimens were used. For the capillary water absorption, prismatic specimens, cut directly from the removed wall pieces, with and without the protective coating system, were also tested. Finally, some small specimens, which were randomly chosen from the pieces that were removed from the wall prototypes, were used for the microstructural tests. In Table 1, the type of specimens, as well as their dimensions and number of tests for each specimen, are presented.

Due to the non-destructive nature of some tests, the same samples were used to characterize different properties before being subjected to any destructive testing. The referred experimental tests were performed for each of the three accelerated aging methods (EN, ETAG, and JM methods, as described in Section 2.1).

### 2.3. Experimental Tests

As shown in Figure 1, two different types of tests were performed: with the removed pieces of aerogel cement-based render and rendering system and the wall prototypes (rendering system only). The removed pieces for the tests were collected from the wall prototypes and the tests were carried out at Instituto Superior Técnico laboratories (DECivil Laboratório de Construção, CQFM chemical laboratory and MicroLab) of the University of Lisbon. The tests on the rendering system applied on the wall prototypes were carried out by the authors in the Laboratory of Building Physics, of the Faculty of Engineering of the University of Porto. The durability tests performed for each referred type are described in the following sections.

#### 2.3.1. Mortar and Protective Coating System

##### Testing Procedures

After producing the specimens, the hardened-state bulk density was evaluated following EN 1015-10 [47], using the geometric method in a hardened state. The volume of the different specimens and their masses were measured using a micrometer (SOMET CZ, Bílina 418 01, Czech Republic) and a two-digit precision weighing scale (KERN & SOHN GmbH, Balingen, Germany), respectively.

**Figure 5:**
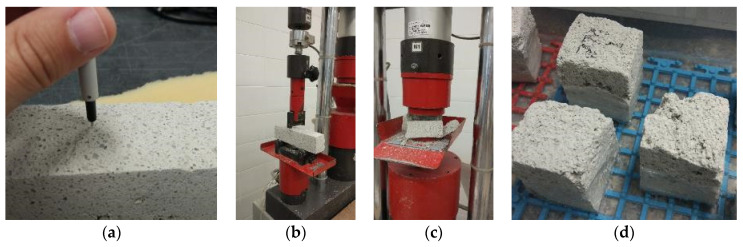
(**a**) Dynamic modulus of elasticity test; (**b**) flexural and (**c**) compressive strength tests; (**d**) capillary water absorption test specimens with sealed faces.

The dynamic modulus of elasticity was determined using GrindoSonic MK5 Industrial equipment (GrindoSonic, 3001 Leuven, Belgium), which measures the resonance frequency of the flexural and torsional vibration modes, according to ASTM (American Society for Testing and Materials) E1876-15 [48]. This equipment also determines the Poisson coefficient of mortar. In Figure 5a, it is possible to see the transducer in contact with a specimen (specimen removed from the wall, after preparation).

The compressive and flexural strengths were determined according to EN 1015-11 [49] procedures using Form + Test (model 505/200/10 DM1) equipment (FORM + TEST Seidner&Co. GmbH, 88499 Riedlingen, Germany). Two different load cells were selected for the compressive and flexural strength tests: 200 kN and 10 kN, respectively. The flexural strength tests were made first, and, with the resulting halves, the compressive strength tests were then carried out. Figure 5b,c shows both tests.

The capillary water absorption test was carried out according to EN 1015-18 [50]. Render specimens with and without the protective coating system were tested. First, the side faces of all specimens were sealed with epoxy paint, promoting a unidirectional water flow (bottom to top). After 24 h, the specimens were dried at 60 ± 5 °C until mass stabilization. The specimens were then conditioned, in a sealed box with silica gel, at room temperature and then placed into a water-containing vessel (at least 5 mm depth) (see Figure 5d). Afterwards, their masses were evaluated at 10 and 90 min, which allowed the determination of the capillary water absorption coefficient (C).

##### Microstructural Testing Procedures

The Brunauer–Emmett–Teller (BET) technique allowed estimating the specific surface area and pore size distribution of the samples, analyzing the N2 adsorption isotherms at 77K [51]. As such, an ASAP 2020 (Micromeritics, Norcross, GA, USA) was used, as the specimens were initially degassed by heating under vacuum, and the adsorbed impurities, gases, or vapors were removed. Then, an estimation of the average size distribution of the mesopores was performed using the Barret–Joyner–Halenda (BJH) model applied to the adsorption branch [52]. However, some care must be taken when evaluating the results, since only the interval 5 < CBET < 150 (CBET: BET constant) is valid [53].

Regarding the MIP testing technique, AutoPore IV 9500 equipment (Micromeritics, Norcross, GA, USA) was used. This equipment allowed to evaluate the pressure versus intrusion data, providing the volume and size distribution using the Washburn equation [54], assessing the open porosity [55,56].

For the two previously described techniques, and to establish pore classification, IUPAC (International Union of Pure and Applied Chemistry) [53] pore classification was followed: micropore (internal diameter < 2 nm), mesopore (between 2 and 50 nm), and macropore (>50 nm). Since the two previous techniques had some overlapping in their measurement ranges, a transitional 100-nm pore diameter [57] was adopted, allowing for a smoother transition [58] between them.

To visualize the microstructure, such as the aspect of pores, hydration products, or even microstructural degradation due to accelerated aging conditions, a scanning electron microscope (SEM) Hitachi S-2400 SEM, (Hitachi, Krefeld, Germany) was used. To prevent alterations of the surface area of the specimens, a palladium film was applied on their surfaces. For this evaluation, two samples of each wall were subjected to three accelerated aging methods and were observed. One of the main limitations related to the SEM images is their purely qualitative nature; therefore, they can be often misleading depending on the chosen sample. Additionally, due to the small size of the sample, there is a great deal of difficulty in the selection of a representative part of the whole material [56]. Nonetheless, in the present work, the SEM images were analyzed together using other microstructural analysis techniques (such as MIP and BET), allowing the interpretation of other measured properties, and complementing each other. These techniques are very useful to characterize the distribution of pores (porosimetry), from nanopores to macropores.

#### 2.3.2. Wall Prototypes

**Figure 6:**
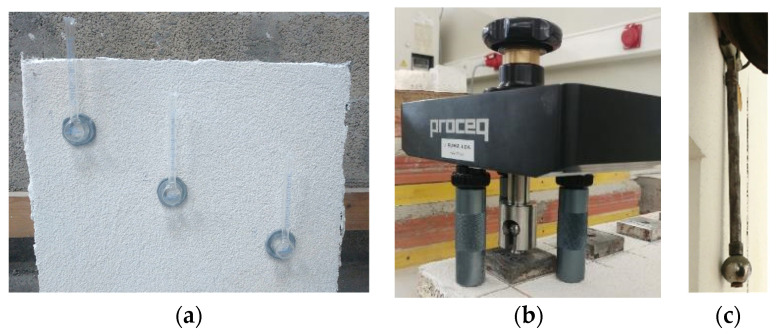
(**a**) Karsten tubes test; (**b**) adhesive strength test; (**c**) impact resistance test.

The liquid water permeability was determined using the Karsten tubes method [59], by placing three L-shaped tubes vertically, as shown in Figure 6a. Each tube had volume graduation, corresponding 10 mL to 10 cm of water. The tubes were bonded to the external surface with silicone and filled until 10 mm, which corresponded to 0 mL scaling. The water level decrease was recorded at 5, 10, 15, 20, 25, 30, 40, 60, and 120 min, as well as 24 h, and the volume of water that penetrated the systems (V_t_) after 1 h and 24 h was calculated.

The adhesive strength (f_u_) was determined based on EN 1015-12 [60] using Proceq DY-216 equipment (Proceq, Schwerzenbach, Switzerland) (see Figure 6b). Stainless steel square pull-heads (50 × 50 mm) were glued to the surface using epoxy resin (3 measurements per specimen). Before fixing the pull-heads, two different pre-cuts were made in each measurement, with controlled cutting depth, to the substrate, to evaluate the adhesive strength between the render and the substrate, and in the render itself, to assess the strength between the render and the protective coating system. Regarding the high disruption of the drilling machine and the low mechanical strength of the thermal mortars [61], the EN 1015-12 approach, where circular pull-heads are used, was not followed.

Impact resistance was determined using EN 13497 [62], using a Martinet Baronnie apparatus, as can be seen in Figure 6c. This test consists of a qualitative evaluation of visual damage due to the impact of a steel ball coupled to a tilting metal arm, which is positioned at 90° from the specimen and then released for impact, with an impact energy of 3 Joules, and three impacts per specimen. In addition to the qualitative assessment, the result of the average value of the diameters of the produced dents (ϕ) was determined, as described in ISO (International Organization for Standardization) 7892 [63]. ETAG 004 [64] also provides a classification of the system into three distinct categories, attesting to its suitability for possible use.

## 3. Results and Discussion

### 3.1. Hardened-State Characterization

For the hardened-state characterization, the test conditions presented average ambient conditions of 20 °C and 60% RH. The laboratory results for the specimens removed from the wall prototypes after the accelerated aging tests are presented in Table 2; for the wall prototypes with the rendering system applied they are presented in Table 3. These results were compared with a specimen (produced in the same way as the aged specimens) that was not submitted to accelerated aging. As such, the non-aged specimen is used as the reference (designated “Ref”).

Analyzing the average bulk density of the different specimens, it is possible to see that there is a significant difference between the three aging methods and the reference (non-aged). This can be due to the application efficiency of the projection machine, which influenced these results (the render presents a heterogeneous constitution, as can be seen in Figure 4a and Figure 5d, by the air void dimensions and distributions).

Regarding the dynamic modulus of elasticity and the Poisson coefficient, only the JM method presented higher values (30% more than the reference and EN and ETAG methods). Regarding the increase in the Poisson’s coefficient after the EN and ETAG methods, some added lateral expansion when compressed was observed, when compared to the reference. However, the obtained dynamic modulus of elasticity was low with these two methods, denoting a significant capacity to deform with substrate movements. This fact could be related to the configuration of the specimens: the EN and ETAG methods were performed in small-scale walls, while the JM method was in the full-scale wall and built-in the rig (that could restrict the movements).

**Figure 7:**
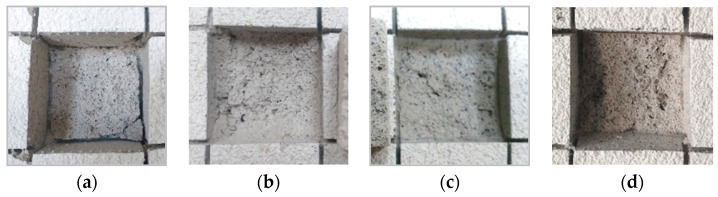
Cohesive fracture patterns: (**a**) Ref (pre-cut until the substrate); pre-cut in the render itself: (**b**) EN; (**c**) ETAG; (**d**) JM.

When analyzing mechanical resistances, it could be seen that both the compressive (f_c_) and flexural (f_t_) strengths decreased in all the accelerated aging methods when compared with the reference. In contrast, adhesive strength (f_u_) increased, in general, after the aging methods, being more significant in the EN and ETAG methods (visible deterioration of the rendering matrix in Figure 7b,c). This fact could be due to the presence of liquid water in both procedures which may contribute to the late hydration of binder particles and, consequently, to an increase in adhesive strength. On the other hand, the JM method presented similar results to the reference (less deterioration of the render—see Figure 7d). Another finding is the higher variability of the results after the aging methods (observed by the higher standard deviation), highlighting the importance of increasing the number of pull-off specimens, as stated by Ramos et al. [65]. Observing the results of the two different pre-cuts, higher adherence values were obtained in the pre-cut performed in the render itself. As observed during the test, the pre-cut was performed until the substrate promoted the weakening of the render, even without aging, as can be seen in Figure 7a. This was especially evident in the specimens using the ETAG method, which contributed to the higher variability of the results. Despite the low adhesive strength, which is expected in these types of thermal mortars, cohesive fractures in the render itself were observed, which were within the admitted values in ETAG 004 [64] (for a thermal multi-layered system with boards insulating materials) and the results obtained by Maia et al. [66] for thermal rendering systems. As discussed by Flores-Colen et al. [67], because of the low weight of aerogel cement-based renders and their vulnerability to thermal variation, the low adhesive strength could be admitted.

**Figure 8:**
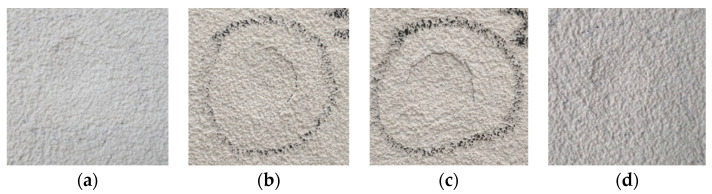
Hard body impact dent: (**a**) Ref; (**b**) after ETAG; (**c**) after EN; (**d**) after JM.

In terms of impact resistance, higher cracking of the coating and dent diameter after the EN and ETAG aging methods were observed (see Figure 8), where the water action contributed to increasing the deterioration; however, these values should be carefully analyzed since the measurement procedure (in millimeters) presented some variation. This fact highlights the importance of qualitative analysis through observation of the caused damages. Since the coating was not penetrated (only surface cracking of the outer layer) the studied system could be classified as Category III according to ETAG 004 [64].

**Figure 9:**
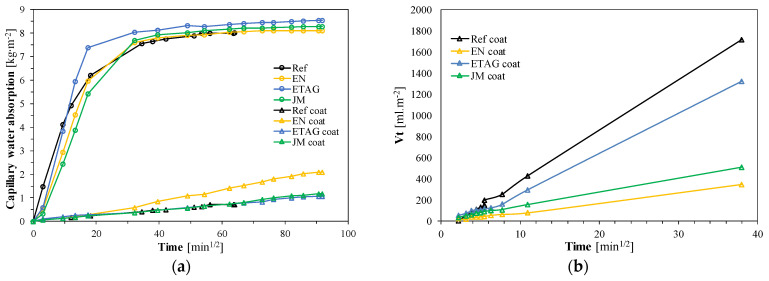
(**a**) Capillary water absorption; (**b**) liquid water permeability absorption.

For the capillary water absorption, both results for coated and uncoated specimens are presented in Figure 9a and for the liquid water permeability of the coated specimens (wall prototype test) in Figure 9b.

For the uncoated specimens, the initial slope was similar for all specimens (see Figure 9a), which is also translated in very similar capillary water absorption coefficients between them (around 0.800 kg∙m^−2^∙min^−1/2^), except for the specimens using the ETAG aging method. These are very high values considering exterior application according to EN 998-1 [68]. However, the uncoated specimens (Ref) could reach saturation earlier. As for the coated specimens, before aging (Ref) and after the ETAG and JM methods, results of around 0.020 kg∙m^−2^∙min^−1/2^ were observed, with the EN method showing a significantly higher value. Regarding the obtained curves (Figure 9a), the ETAG and JM methods showed similar initial slopes, while the EN method showed a higher slope. In addition, after applying the EN method, stabilization was reached later, which indicated a more considerable influence on the coating integrity. The EN method included infra-red radiation, which can promote an increase in degradation of the coating, supporting these findings.

On the other hand, the results of the liquid water permeability, performed in the wall prototypes, were the opposite of those previously analyzed, which highlighted the importance of reducing the interference in the collection of specimens from the walls to achieve more reliable results. Nevertheless, the aging methods promoted a reduction in the absorbed water volume. Over time, the difference between the reference and the aging methods became higher. It was possible to observe that the studied system promoted a reduction in liquid water absorption in both the EN and ETAG methods, contrary to the system tested by Maia et al. [66] (with different thermal renders, without aerogel) where it increased. This fact evidences that the new aerogel cement-based render could promote an improvement in the behavior of thermal-enhanced façades in terms of liquid water absorption regarding the tested aging methods, which could be related to a rearrangement of the microstructure.

### 3.2. Microstructural Characterization

As previously stated, to obtain valid results with the N2 adsorption/desorption isotherms at 77K (BET) technique, the value of the CBET parameter, when analyzing the nitrogen isotherms using the BET equation, should be between 5 and 150 [53]. Table 4 shows the test results for all the test specimens. It is also possible to see that all tests complied with the CBET requisites. The reference, as previously presented, corresponds to the same render formulation applied to a lightweight concrete block wall, but without accelerated aging procedures. When the results are compared with the silica aerogel performance studied by Júlio et al. [57], it can be seen that this lightweight aggregate has a significant influence on the overall performance of the composites, since it represents, as previously indicated, ~70% of the total volume. The accelerated aging methods did not produce a significant impact on the microstructure of the render, presenting similar values between them. However, the specific surface area (S) reduced after ageing, while the total pore volume (V_p_) remained similar, indicating that the methods increased the pore size [69].

**Figure 10:**
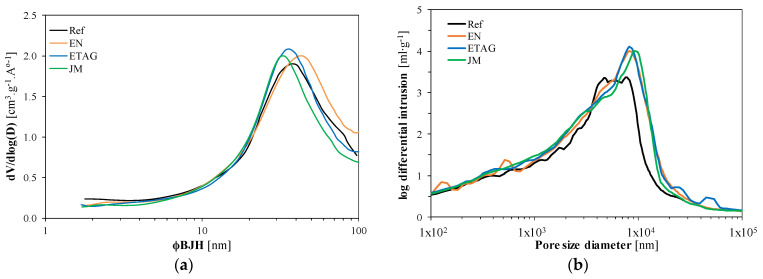
(**a**) BJH mesopore size distribution; (**b**) average distribution of the dimension of macropores obtained through MIP.

To assess the mesopore size distribution, BJH analysis of the adsorption branch of the isotherms was performed. From Figure 10, it is possible to observe mesopores from 2 nm onward, with a maximum size achieved between 30 and 50 nm, right at the limit of mesopore classification. In addition, specimens subjected to accelerated aging methods increased the number of pores with a higher diameter (comparing the reference with the aging methods) explaining the reduction in the specific surface area. Nonetheless, the overall behavior was very similar.

**Figure 11:**
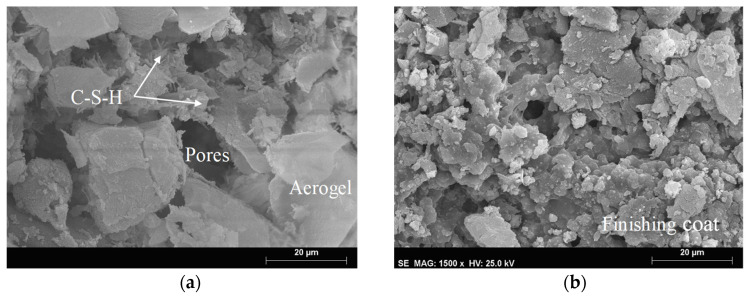
General aspect of the microstructure before aging: (**a**) aerogel cement-based render; (**b**) finishing coat.

**Figure 12:**
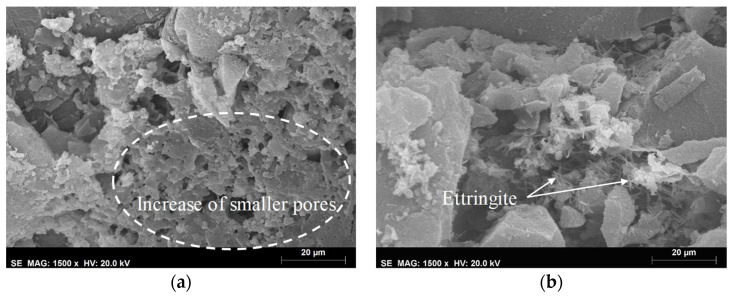
Microstructure of the render after the: (**a**) EN method; (**b**) JM method.

**Figure 13:**
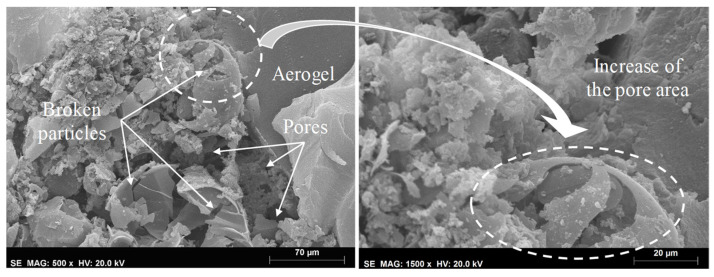
Microstructure of the render after the ETAG method.

Table 4 presents the results obtained through the MIP technique after aging. Regarding the specimens subjected to accelerated aging, an increase in the total pore area was also found. An interesting behavior, after applying the EN method was found, where, although there was a decrease in open porosity comparing to the reference, the total pore area increased, which can indicate a higher number of smaller pores [70]. Although the MIP technique is often used to characterize the pore structure, some degradation, caused by the pressure applied to the mercury, may occur. This fact could be due to the low mechanical resistance of these aerogel-based mortars, increasing their porosity values. Nonetheless, as observed by Feldman [71] and Aligizaki [69], and from the SEM images (Figure 11, Figure 12 and Figure 13), these renders presented high porosity.

As can be seen in Figure 10b, the maximum pore size diameter was between 4 × 10^3^ and 1 × 10^4^ nm, with the reference presenting a plateau, and the other specimens showing the increase in higher pore size diameters (mainly between 6 × 10^3^ to 2 × 10^4^ nm). Considering this difference in the macropores, and the work developed by Zeng et al. [72], the increase can be related to pores, voids or cracks, or irregularities present on the specimen surfaces. In this case, these results can be related to the accelerated aging methods and their influence on the microstructure of the uncoated render, which may influence its mechanical and physical performances.

### 3.3. Discussion of the Results

The application of the aerogel cement-based render presents some variability due to the early stage of development, which can lead to some performance heterogeneity; namely, the water adjustment process on the mechanical projection machine and its operation. However, this procedure reproduces the problems and constraints that occur in situ, which highlights the importance of this work. The same rendering coating system is also used for comparison in several aging approaches.

Despite the great caution regarding the collection of individualized specimens from the wall prototypes, mechanical manipulation (through sawing and grinding) can influence their performance. However, any other method allows obtaining specimens for testing after being applied to a wall. Despite this, the conditions were the same for all the specimens.

Observing Figure 11, differences between the microstructure of the aerogel cement-based render and the finishing coat could be identified, which corroborated the obtained results. Despite the gain in mechanical resistance due to the hydration of cement paste (formation of C-S-H), the high open porosity compromised the mechanical behavior and water absorption. In this way, the outer layer (finishing coat) is of great importance to reducing water penetration, acting as a protective barrier, as shown in the capillary (EN 1015-18 method) and liquid water absorption (Karsten tubes method) tests. Additionally, the finishing coating allowed to protect the aerogel cement-based render against impacts, which were observed in the hard body impact test.

The mechanical and physical properties of the specimens subjected to accelerated aging mainly showed a decrease in performance. An accurate analysis of the porous structure allowed the identification of some causes of the loss of performance. As can be seen in Figure 10, accelerated aging promoted an increase in mesopores and, mainly, macropores. This increase was greater with the EN and ETAG methods, due to the water penetration and freeze-thawing procedures that promoted material expansion and contraction, leading to higher pore dimension and cracking, as already stated by Maia et al. [66] and Zeng et al. [72].

Observing Figure 12a, it is possible to see the increase in pores with smaller dimensions after applying the EN method, which is in line with the results obtained with the MIP technique. In both BJH and MIP analyses, an increase in pore size was observed, especially after using the ETAG method. Since this method comprises an aggressive aging procedure (higher number of freeze-thaw cycles), the microstructure presented several broken particles (as shown in Figure 13), which promoted an increase in pores, voids and/or cracks, corroborating the obtained results. As long as the porosity influences the mechanical resistance, the decrease in compressive strength could be associated with the increase of the pores. Although this loss of resistance, a densification of the matrix, after aging, can be seen in Figure 12 from the bonds created between the different materials and the ettringite crystals. This fact could be attributed to the late hydration of the cement, after the initial hardening, contributing to an increase in adhesive strength [73].

The reduction in liquid water absorption after aging procedures could also be related to a reduction in the capillary network (an increase of the densification of the matrix possible due to late hydration of the binder and, consequently, a rearrangement of the porous structure). Despite the increase in the pore size, the microstructural rearrangement may influence those results. The required conditioning after aging was correctly performed, being the specimens dried.

## 4. Conclusions

The adaptation of different accelerated aging methods allowed the evaluation of long-term performance of a new aerogel cement-based render and its protective coating in the context of distinct combined degradation mechanisms, such as heating–freezing and humidification-freezing, freeze-thawing and adapted heat-cold cycles. However, handling of the small-scale prototypes should be carefully performed, due to the fragility of this rendering system, to avoid external disturbances to the methods and, consequently, to the aged properties. In addition, the application of the render by mechanical spraying, which is the adopted application method for these types of lightweight mortars, allowed a more realistic evaluation of the in situ conditions.

A general decrease in the mechanical resistance, except for the adhesive strength, was observed. Both flexural and compressive strengths were lower after aging (around 0.04 and 0.09 MPa, respectively), especially for heating-freezing plus humidification-freezing and freeze-–thawing methods. However, this reduction was followed by a decrease in the dynamic modulus of elasticity (≈19.5 MPa), which indicated a higher capacity of the render to deform with substrate movements. This, together with the increase in the Poisson’s ratio (0.3), may contribute to reducing the occurrence of cracking. The water action promoted the late hydration of the cement paste, contributing to the densification of the matrix and, consequently, an increase in the adhesive strength (more than doubles comparing to the non-aged specimen). Despite the low adhesive strength, cohesive fractures in the render itself were obtained. These values could be accepted, considering the low weight of aerogel cement-based renders and their vulnerability to thermal gradients. Another relevant aspect was the reduction in liquid water absorption regarding the tested aging methods, which could be related to a rearrangement of the microstructure; for example, densification and an increase in smaller pores in the matrix of the aerogel cement-based render, due to the liquid water action.

The application of the protective coating contributes to decreasing the deterioration of the system as a whole, especially regarding the high decrease in capillary water absorption (around four times lower). This is of great importance since the capillary water absorption coefficients were bigger than 0.75 kg∙m^−2^∙min^−1/2^, and were very high values considering the exterior application according to EN 998-1.

Regarding the aging methods and the related degradation mechanisms, the heating–freezing plus humidification–freezing and freeze-thawing methods promoted a higher variation in the tested properties than the heat–cold cycle, due to the greater severity of the aging procedures, which includes freeze-thaw. This mechanism combined with temperature gradients, including infra-red radiation (which is the case with heating–freezing), could promote an increase in the degradation of the protective coating, observed by the higher period to reach stabilization (to water saturation).

In summary, durability highly depends on the porous structure, which, consequently, affects liquid water absorption and mechanical strength. Care should be taken when an increase in the adhesive strength is observed since the values are still very low (≈0.13–0.16 MPa), despite cohesive ruptures. However, these values are within the obtained range for lightweight façade systems, such as thermal renders and ETICS. The durability assessment of thermal rendering systems, such as aerogel-based rendering systems, is of high importance since it allows an analysis of the main degradation mechanisms that will cause relevant degradation. In that way, the combination of freeze-thaw and temperature gradients applied to full-scale specimens, avoiding handling and scale factor influence, will allow a more reliable durability assessment of aerogel-based rendering systems.

## Figures and Tables

**Table 1:** Characteristics and number of specimens for each experimental test.

Shape of Specimens	Test	Dimension [mm × mm × mm]	Number of Tested Specimens per the Aging Method and Test
Prismatic	BD; E_d_; ν; f_t_; f_c_	160 × 40 × 40	3
Prismatic no coat	C	50 × 50 × 42	4
Prismatic coated	C	50 × 50 × 54	4
Small pieces	BET; MIP; SEM	random	3

Caption: BD—bulk density; E_d_—dynamic modulus of elasticity; ν—Poisson’s coefficient; f_t_—flexural strength; f_c_—compressive strength; C—capillary water absorption; BET—Brunauer–Emmett–Teller technique; MIP—mercury intrusion porosimetry; SEM—scanning electron microscope.

**Table 2:** Experimental results of the removed pieces.

Method	BD ± SD [g·cm^−3^]	E_d_; ν [MPa]	f_t_ ± SD [MPa]	f_c_ ± SD [MPa]	C_uncoated_ ± SD [kg∙m^−2^∙min^−1/2^]	C_coated_ ± SD [kg∙m^−2^∙min^−1/2^]
Ref	0.178 ± 0.006	26.0; 0.23	0.070 ± 0.008	0.147 ± 0.004	0.850 ± 0.030	0.020 ± 0.004
EN	0.165 ± 0.005	18.5; 0.30	0.042 ± 0.004	0.080 ± 0.003	0.755 ± 0.247	0.030 ± 0.010
ETAG	0.173 ± 0.009	20.5; 0.30	0.042 ± 0.014	0.105 ± 0.012	0.900 ± 0.254	0.027 ± 0.012
JM	0.178 ± 0.009	33.7; 0.31	0.059 ± 0.007	0.117 ± 0.013	0.780 ± 0.212	0.017 ± 0.012

Caption: BD—bulk density; E_d_—dynamic modulus of elasticity; ν—Poisson’s coefficient; f_t_—flexural strength; f_c_—compressive strength; C—capillary water absorption; SD—standard deviation.

**Table 3:** Experimental results of the wall prototypes (rendering system).

Method	f_u_ (Substrate) ± SD [MPa]	f_u_ (Render) ± SD [MPa]	ϕ ± SD [mm]	V_t_ (1 h) ± SD [ml.m^−2^]	V_t_ (24 h) ± SD [ml.m^−2^]
Ref	0.057 ± 0.006	0.063 ± 0.006	23.29 ± 1.805	254.65 ± 3.473	1716.52 ± 0.651
EN	0.130 ± 0.020	0.130 ± 0.017	30.82 ± 6.244	66.02 ± 0.136	348.96 ± 0.046
ETAG	0.087 ± 0.075	0.160 ± 0.082	30.06 ± 6.041	160.33 ± 0.953	1322.75 ± 0.021
JM	0.040 ± 0.022	0.070 ± 0.010	22.87 ± 1.577	108.46 ± 0.545	509.30 ± 0.097

Caption: f_u_—adhesive strength; ϕ—dent diameter (hard body impact); V_t_—water volume (liquid water permeability test); SD—standard deviation.

**Table 4:** BET and MIP results.

Method	BET					MIP			
	m [g]	S [m^2^∙cm^−1^]	C_BET_	V_P_ [cm^3^∙g^−1^]	Φ_BJH_ [nm]	CA [Degree]	m [g]	Total Pore Area [m^2^∙g^−1^]	ε [%]
Reference	0.5351	281	21	1.24	16.17	140	0.1833	61.6	83.6
EN	0.2259	270	22	1.25	16.02	140	0.1665	68.8	81.5
ETAG	0.2916	264	22	1.21	15.97	140	0.1586	67.7	85.7
JM	0.2051	252	22	1.20	16.18	140	0.1640	63.2	86.2

Caption: m—specimen mass; S—specific surface area; C_BET_—BET constant; V_P_—total pore volume; Φ_BJH_—mean mesopore diameter obtained by the BJH method; CA—contact angle; ε—open porosity.

## Data Availability

The data presented in this study are available on request from the corresponding author.
